# Inbreeding levels in Northeast Brazil: Strategies for the prospecting of new genetic disorders

**DOI:** 10.1590/S1415-47572010005000020

**Published:** 2010-06-01

**Authors:** Silvana Santos, Fernando Kok, Mathias Weller, Francisco Rennan Lopes de Paiva, Paulo A. Otto

**Affiliations:** 1 Centro de Estudos do Genoma Humano, Departamento de Genética e Biologia Evolutiva, Instituto de Biociências, Universidade de São Paulo, São Paulo, SPBrazil; 2 Departamento de Biologia, Centro de Ciências da Saúde, Universidade Estadual da Paraíba, Campina Grande, PBBrazil; 3 Departamento de Neurologia, Hospital das Clínicas, Faculdade de Medicina, Universidade de São Paulo, São Paulo, SPBrazil; 4 Prefeitura do Município de Martins, Martins, RNBrazil; 5 Departamento de Genética e Biologia Evolutiva, Instituto de Biociências, Universidade de São Paulo, São Paulo, SPBrazil

**Keywords:** Inbreeding levels, genetic drift, geographic isolation

## Abstract

A new autosomal recessive genetic condition, the SPOAN syndrome (an acronym for spastic paraplegia, optic atrophy and neuropathy syndrome), was recently discovered in an isolated region of the State of Rio Grande do Norte in Northeast Brazil, in a population that was identified by the IBGE (Brazilian Institute of Geography and Statistics) as belonging to the Brazilian communities with the highest rates of “deficiencies” (Neri, 2003), a term used to describe diseases, malformations, and handicaps in general. This prompted us to conduct a study of consanguinity levels in five of its municipal districts by directly interviewing their inhabitants. Information on 7,639 couples (corresponding to about 40% of the whole population of the studied districts) was obtained. The research disclosed the existence of very high frequencies of consanguineous marriages, which varied from about 9% to 32%, suggesting the presence of a direct association between genetic diseases such as the SPOAN syndrome, genetic drift and inbreeding levels. This fact calls for the introduction of educational programs for the local populations, as well as for further studies aiming to identify and characterize other genetic conditions. Epidemiological strategies developed to collect inbreeding data, with the collaboration of health systems available in the region, might be very successful in the prospecting of genetic disorders.

## Introduction

In the 1950's and 1960's, Freire-Maia and his collaborators, as well as other authors, conducted a number of studies on inbreeding levels of Brazilian populations. The overall mean value of the frequency of consanguineous marriages in Brazil was estimated to be 4.8%, corresponding to an average inbreeding coefficient F = 0.0023. As expected, the lowest inbreeding levels were detected in the southern states, whereas in some states of Northeast Brazil these rates varied from 6% to 12% (Freire-Maia, 1957, 1958; Freire-Maia and Freire-Maia, 1961; see also Salzano and Freire-Maia, 1967; Freire-Maia, 1968, 1989, 1990, for summaries and updates of these studies).

Recently, members of our group described a new neurological autosomal recessive syndrome, known as SPOAN, affecting families living in the state of Rio Grande do Norte in Northeast Brazil (Macedo-Souza *et al.*, 2005, 2009). Since most of the affected subjects were inbred, we became interested not only in evaluating the consanguinity rates in the region, but also in developing a project to study the consequences of these rates on morbidity.

## Methods

The study was developed with the participation of agencies involved in programs for the training of health agents [“Programa de Agentes Comunitários de Saude (PACS)”] and for promoting family health [“Programa de Saúde da Família (PSF)”], in five municipal districts of the state of Rio Grande do Norte (São Miguel, Pilões, Riacho de Santana, Serrinha dos Pintos, and Olho d'Água do Borges), all of them recently identified by IBGE as belonging to the 50 Brazilian communities with the highest rates of “deficiencies” (Neri, 2003).

**Figure 1:**
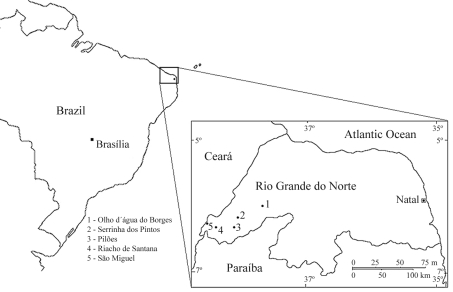
Surveyed villages in western Rio Grande do Norte, Northeast Brazil.

These five municipal districts are located in a relatively isolated rural region, 350 to 400 km away from the state capital Natal (Figure 1). According to IBGE census figures, the population encompassed by the present study consisted, in 2007, of 39,054 inhabitants distributed as follows: Serrinha dos Pintos, 4,360; Riacho de Santana, 4,292; Pilões, 3,381; Olho d'Água do Borges, 4,442; and São Miguel, 22,579.

The data collection to determine the consanguinity levels was performed by community agents of the PACS program, under the supervision of a team of nurses of the PSF program and the scientific staff involved directly in the present study. In a two-day course, health agents and nurses were given the pertinent instructions and training as to interviewing the married couples of the communities and filling out a questionnaire with information regarding the type of possible parental consanguinity, the number of children per couple, as well as diseases, malformations, handicaps and other health problems presented by close relatives. Part of the data was collected as a pilot study, with the aim of clarifying and correcting any mistakes which might have been made by the agents trained to participate in the field study.

The degree of biological relationship of the couples was obtained by direct interviews performed by the health agents and classified according to the offspring's corresponding F values (see footnote of Table 1 for details). The average inbreeding coefficient for each population aggregate was evaluated by weighting the individual F values by their estimated frequencies.

In three municipalities (Serrinha dos Pintos, Pilões and Riacho de Santana), information on all couples was obtained by interviewing one or both members of each couple (1,347, 980 and 798 couples, respectively, grossly corresponding to 62, 46 and 47% of the total respective populations). In Olho d'Água do Borges and São Miguel, only a sample of individuals was interviewed (providing information on 828 and 3,692 couples, respectively, in the two municipalities, corresponding to about 37 and 33% of their total populations), after a random selection of homes to be visited (all homes in a series of randomly chosen streets were visited until the above figures were reached).

## Results

Out of a total of 39,054 inhabitants (number estimated by the IBGE 2007 census), information on 7,639 couples (corresponding to about 40% of the total population) was personally obtained through direct interview of one or both members of each couple. Considering that the average number of children per married couple in the region is low, presently ranging from two to three children, it can be safely concluded that the data of most families were included in the present study.

Validation of the data collected by the health agents was performed using the following method: members of the scientific staff visited some of the interviewed families in the municipal district of Pilões for a second time, and 1/8 of the interviews made by the health agents were repeated, the corresponding questionnaires being filled out again, in order to be compared with the first ones. A negligible error (corresponding to less than 3% of the couples) was detected in the description of the true degree of biological relationship.

Table 1 shows the estimates of the relative frequencies of different types of consanguineous unions in the five districts, the average coefficients of inbreeding for each locality and for the set of all five localities.

## Discussion

Of the five districts surveyed here, Olho d'Água do Borges, located beside a main highway and therefore characterized by a population with a higher mobility rate, presented, as expected, the lowest average frequency of consanguineous marriages (about 9%) and the smallest average inbreeding coefficient (F = 0.0032). Pilões, a population aggregate founded more recently by families originating from different communities and settlements of the region, presented the second lowest inbreeding level (about 12% of consanguineous couples, F = 0.0036). Riacho de Santana (F = 0.0049) and São Miguel (F = 0.0073) showed similar average frequencies of consanguineous unions, about 19%. In Serrinha dos Pintos, more than 30% of all couples were constituted by related individuals (F = 0.0095), a fact that can be explained by the relative geographic isolation of its population, living in a highland region (on average 400 m above sea level) in the western part of the state of Rio Grande do Norte.

While the frequency of consanguineous marriages is at its lowest in the state of São Paulo in Southeast Brazil (less than 1% of all unions), rates as high as 6 to 12% have been recorded in rural areas of Northeast Brazil (Freire-Maia, 1957). In most of the surveyed villages reported here, the inbreeding rates were much higher, some of them ranging from 20% to 30%. The results of the present study clearly indicate that the consanguinity levels have not fallen in Northeast Brazil. This is not surprising, however, because, in contrast to other parts of Brazil, this region did not undergo any significant economic and geopolitical changes in the last 50 years.

In Serrinha dos Pintos, where 32.5% of all marriages took place between biologically related individuals, the continuation of this practice is directly associated with the manifestation of at least one monogenic autosomal recessive disease, the already-mentioned SPOAN syndrome, whose gene increased in frequency in its population probably due to random genetic drift. In the specific case of this syndrome, the habit of consanguineous marriages has remained unchanged for generations. The individuals of this population are unaware of the fact that there is an association between the practice of consanguineous marriages and the rate of manifestation of genetic diseases (Santos and Bizzo, 2005; Santos, 2006).

We suggest that the methodology developed in the present study should be applied for analyzing other populations of Northeast Brazil, in order to ascertain their consanguinity levels and also to possibly identify other genetic conditions expressed in these populations, due to the combined effects of genetic drift and inbreeding. The data so collected should be used to inform the families through educational programs on the genetic risks due to unions between close relatives.

## Figures and Tables

**Table 1:** Relative frequencies of consanguineous marriages (all values in percentage).

	TS	PD	P1	P2	P3	P+	Total C	NC	F
SP	0.15	0.45	8.24	7.05	6.38	10.24	32.52	67.48	0.0095
RS	0.00	0.20	4.08	4.08	3.67	7.35	19.39	80.61	0.0049
Pi	0.00	0.38	3.13	2.38	2.01	3.88	11.78	88.22	0.0036
OA	0.12	0.61	2.31	1.58	1.22	3.04	8.88	91.12	0.0032
SM	0.68	0.73	5.93	3.82	2.76	5.04	18.96	81.04	0.0073
Total	0.37	0.56	5.42	4.03	3.27	5.92	19.57	80.43	0.0066

TS = uncle-niece (or aunt-nephew) pairs (F = 1/8); PD = couples of double first cousins (F = 1/8); P1 = first cousins (F = 1/16); P2 = first cousins once removed (F = 1/32); P3 = second cousins (F = 1/64); P+ = couples with far and unclassified biological relationship, with an arbitrarily assigned value of F = 1/128); Total C: all consanguineous unions; NC: non-consanguineous unions in the municipal districts SP = Serrinha dos Pintos, Pi = Pilões, RS = Riacho de Santana, OA = Olho d'Água do Borges, SM = São Miguel and in the set of five municipalities (Total). F: average inbreeding coefficient estimated for each locality and for the set of five districts.
